# Post-acute sequelae of COVID-19 and longitudinal antibody levels in a community-based cohort

**DOI:** 10.1371/journal.pone.0291259

**Published:** 2023-09-08

**Authors:** Noa Kopplin, Angie Garcia, Annika Reczek, Kate Wilkinson, Sruthi Yekkaluri, Caitlin C. Murphy, Jasmin Tiro, Alagar R. Muthukumar, Andrew Masica, Amit G. Singal

**Affiliations:** 1 University of Texas Southwestern Medical Center, Dallas, TX, United States of America; 2 Texas Health Resources, Fort Worth, TX, United States of America; Bay Area Hospital, North Bend Medical Center, UNITED STATES

## Abstract

**Background:**

Coronavirus disease 2019 (COVID-19) infection invokes variable immune responses and poses a risk of post-acute sequelae SARS-CoV-2 infection (PASC) symptoms; however, most data on natural history are derived from patients with severe infection. Further data are needed among patients with mild infection, who comprise most cases.

**Methods:**

The Dallas Fort-Worth (DFW) COVID-19 Prevalence Study included 21,597 community-dwelling adults (ages 18–89) who underwent COVID-19 PCR and anti-nucleocapsid antibody testing between July 2020 and March 2021. We invited participants with positive COVID-19 results (cases) and a subset with negative results (controls), matched on age, sex, race/ethnicity, and ZIP code, to complete a follow-up questionnaire for PASC symptoms and repeat anti-nucleocapsid testing, and anti-spike antibody testing between July and December 2021.

**Results:**

Of 3,917 adults invited to participate, 2260 (57.7%) completed the questionnaire– 1150 cases and 1110 controls. Persistent symptoms were reported in 21.1% of cases, with the most common being shortness of breath, fatigue, and loss of taste or smell. Among 292 cases with asymptomatic infection, >15% reported new fatigue and 8–10% reported new loss of taste/smell, myalgias, or headache. Median anti-nucleocapsid levels in cases decreased from 3.5U to 0.7U over a median follow-up of 8.6 months. Anti-spike antibody levels at 6–7 months post-vaccination in cases were similar to that of controls.

**Conclusions:**

More than 1 in 5 patients with COVID-19 infection, including those with mild infection, reported persistent symptoms during follow-up. Both nucleocapsid and spike protein antibody levels decreased within six months following a COVID-19 infection and vaccination.

## Background

Since the first reported case of severe acute respiratory syndrome coronavirus 2 (SARS-CoV-2) in December of 2019, there have been 657 million confirmed cases and over 6.5 million deaths worldwide as of December 2022 [1]. COVID-19 extends beyond a respiratory virus and is now understood to be a multisystem disease with constitutional, cardiopulmonary, neuropsychiatric, and cognitive symptoms [2–4]. A high proportion of patients have persistent symptoms months after an acute infection and develop long term effects [2,5,6]. A variety of post-acute sequelae of SARS-CoV-2 infection (PASC) symptoms have been identified, including residual impairments in multi-organ function, particularly pulmonary, cardiac, psychological, and neurological sequelae [7,8].

Most studies of PASC have focused on patients with a history of severe infection or older, higher-risk adults with comorbidities. Fewer data are available for patients with mild disease, younger adults, and racial/ethnic minorities–populations who account for the majority of SARS-CoV-2 cases in the U.S [9–12]. There is a need for further data from more diverse, large and well-characterized cohorts to better understand the health-care burden and the expected outcomes for patients with PASC.

Today, the widespread use of vaccines, testing, and various public health strategies have helped to reduce the spread of SARS-CoV-2 [13–18]. COVID-19 vaccines have been particularly pivotal in curtailing the effects of the infection by reducing severe disease, hospitalization, and death [19–23]. Studies have established that immunity from vaccines and natural infection decreases over time [24–29], and immunity in previously infected persons is superior to that of immunity acquired from two doses of vaccine [26,30]. Previous findings also suggest that population-level immunity will likely increase over time through a combination of widespread vaccination and breakthrough infections with SARS-CoV-2 variants, but further data are still needed to understand the long-term immune response and how it correlates with protection from infection [31]. Accordingly, we characterized PASC symptoms and assessed changes in antibody levels over time in a diverse urban population.

## Methods

### Study population

As previously described, the Dallas-Fort Worth (DFW) COVID-19 Prevalence Study recruited 21,597 community-dwelling adults (ages 18–89) between July 2020 and March 2021 to estimate the prevalence of COVID-19 infection in Dallas and Tarrant counties [32]. Prior to completing COVID-19 PCR and antibody testing, all participants completed a baseline 15-minute questionnaire collecting demographics (age, sex, race/ethnicity, ZIP code of residence), health conditions, socioeconomic factors (education, insurance status, employment, status), COVID-19 symptoms, and attitudes and behaviors regarding COVID-19. The initial phase of the study concluded in March 2021, prior to widespread availability of vaccines to the public in Dallas and Tarrant counties.

Herein, we report results of the longitudinal component of the DFW COVID-19 Prevalence Study, which was conducted from May 2021 to December 2021 among a subset of participants. We invited all participants with positive COVID-19 PCR and/or antibody testing at baseline (cases) and selected participants with negative PCR and antibody testing (controls), matched on age +/- 5 years, sex, race/ethnicity, ZIP code and neighborhood socioeconomic status (based on proportion of low SES addresses in the census block group) to complete a follow-up questionnaire, anti-spike antibody testing, and repeat anti-nucleocapsid testing [32].

### Data collection

Selected participants were emailed an invitation between May 2021 and November 2021 to complete a follow-up questionnaire about long-term effects of COVID-19 and schedule an appointment for antibody testing. The invitation was sent in either English or Spanish, depending on language preference to the baseline questionnaire. Non-responders were sent two reminder messages. The 15-minute questionnaire could be completed by smartphone, tablet, or computer, and assessed subsequent COVID-19 infections, vaccination status (including month of vaccination), comorbid health conditions, and any new or persistent symptoms (**S1 File**).

The questionnaire included six validated instruments measuring depression, anxiety, cognitive function, autonomic dysfunction, fatigue, and functional activity status. Patient Health Questionnaire (PHQ)-2 and Generalized Anxiety Disorder (GAD)-2 both include two questions to screen for depression and anxiety, respectively. Each scale ranges from 0 to 6 with scores >2 indicating likely depression or anxiety, respectively [33,34]. The Functional Assessment of Chronic Illness Therapy (FACIT)-Fatigue scale v4 is a 13-item tool that measures fatigue during daily activities, with higher scores indicating less fatigue [35]. The Cognitive Function Instrument is a 14-item scale, ranging from 0–14, with higher scores indicating cognitive complaints [36]. Finally, the Duke Activity Status Index is a 12-item scale, in which each question is assigned a weight based on metabolic cost and scores range from 0–58.2 points [37]. Participants identify each activity they can perform, and higher scores indicate better functional capacity.

After completing the questionnaire, participants scheduled a lab appointment to test anti-nucleocapsid and anti-spike protein antibody levels. Anti-nucleocapsid antibodies reflect a history of natural infection (or receipt of live attenuated vaccines, which were not available at the time of this study), whereas anti-spike antibodies can reflect either natural infection or vaccination. Anti-nucleocapsid and anti-spike protein IgG antibodies were analyzed using the Abbott Alinity I platform, with index values of 1.4 and 50, respectively, as thresholds for a positive result (3). Within two weeks of completing antibody testing, results and a $20 gift code were mailed to each participant. All participants provided written informed consent for participation in the study. The Institutional Review Board of UT Southwestern Medical Center approved the study.

### Statistical analysis

We compared characteristics of cases and controls using Chi2 tests, as well as the proportion reporting COVID-related symptoms at baseline and follow-up. We also described prevalence of symptoms among the subgroup of controls with subsequent COVID-19 infection. We next described longitudinal trends in anti-nucleocapsid and anti-spike antibody levels at baseline and follow-up, stratified by case/control status and subsequent vaccination status (ever and by date of first vaccine), using box and whisker plots. We also conducted linear mixed effects regression analysis examining the association between case-control status and anti-nucleocapsid levels after adjusting for age, sex, and vaccination status. All statistical analyses were performed using SAS 9.4(SAS Institute Inc., Cary NC), with statistical significance defined as p<0.05.

## Results

### Participant characteristics

Of 3,917 individuals invited to participate, 2260 (57.7%) completed the follow-up questionnaire–including 1150 cases with prior COVID-19 infection and 1110 controls without COVID-19 infection. The median time from baseline to follow-up questionnaires was 8.3 (IQR 6.8–9.2) months. **Table 1** summarizes characteristics of the participants who completed the follow-up questionnaire. Most participants were aged 45–64 years old, majority were female, and three-fourths identified as non-Hispanic White. Most participants resided in high and medium socioeconomic neighborhoods, reported having a four-year college degree, and had health insurance coverage. The majority of participants reported being vaccinated, including 85.4% of cases and 97.4% of controls. The most common vaccine was Pfizer (56.7%), followed by Moderna (38.7%), and Johnson and Johnson (4.4%). The median time from vaccination to follow-up questionnaire was 6 months (IQR 5–8 months). Given differential response rates to the questionnaire between cases and controls (61.0% and 55.7%, respectively), the latter group was significantly older and had a higher proportion of females, non-Hispanic Whites, and persons with a college degree.

**Table 1:** Characteristics of patients who completed questionnaire.

	Cases (n = 1150)	Controls (n = 1110)	p-value**
Age 18–24 years 25–44 years 45–64 years 65–89 years	40 (3.5%) 444 (38.6%) 510 (44.3%) 156 (13.6%)	18 (1.6%) 372 (33.5%) 539 (48.6%) 181 (16.3%)	0.001
Sex (% male)	377 (32.8%)	254 (22.9%)	<0.001
Race/ethnicity Non-Hispanic White Non-Hispanic Black Hispanic Asian Non-Hispanic other	752 (65.4%) 94 (8.2%) 223 (19.4%) 60 (5.2%) 21 (1.8%)	942 (84.9%) 32 (2.9%) 107 (9.6%) 24 (2.2%) 5 (0.5%)	<0.001
Household size 1–2 adults, without children 1–2 adults, with children 3+ adults, without children 3+ adults, with children	534 (46.4%) 313 (27.2%) 164 (14.3%) 126 (11.0%)	633 (57.0%) 288 (25.9%) 114 (10.2%) 63 (5.7%)	<0.001
Education Less than high school High school or equivalent Some college College degree	9 (0.8%) 85 (7.4%) 218 (18.9%) 828 (72.0%)	4 (0.4%) 23 (2.1%) 164 (14.8%) 915 (82.4%)	<0.001
Socioeconomic status (SES)* Low SES Medium SES High SES	67 (5.8%) 480 (41.7%) 576 (50.1%)	73 (6.6%) 423 (38.1%) 609 (54.9%)	0.09
Health Insurance No Yes	98 (8.5%) 1049 (91.5%)	46 (4.2%) 1061 (95.8%)	<0.001

* Neighborhood socioeconomic status (SES) was assigned based on the estimated proportion of low SES addresses in the county-specific census block group, which was defined as a function of the per capita income and percent of owner-occupied housing units.

** Characteristics of cases and controls were compared using Chi2 tests.

### COVID-19 symptoms

**Fig 1:**
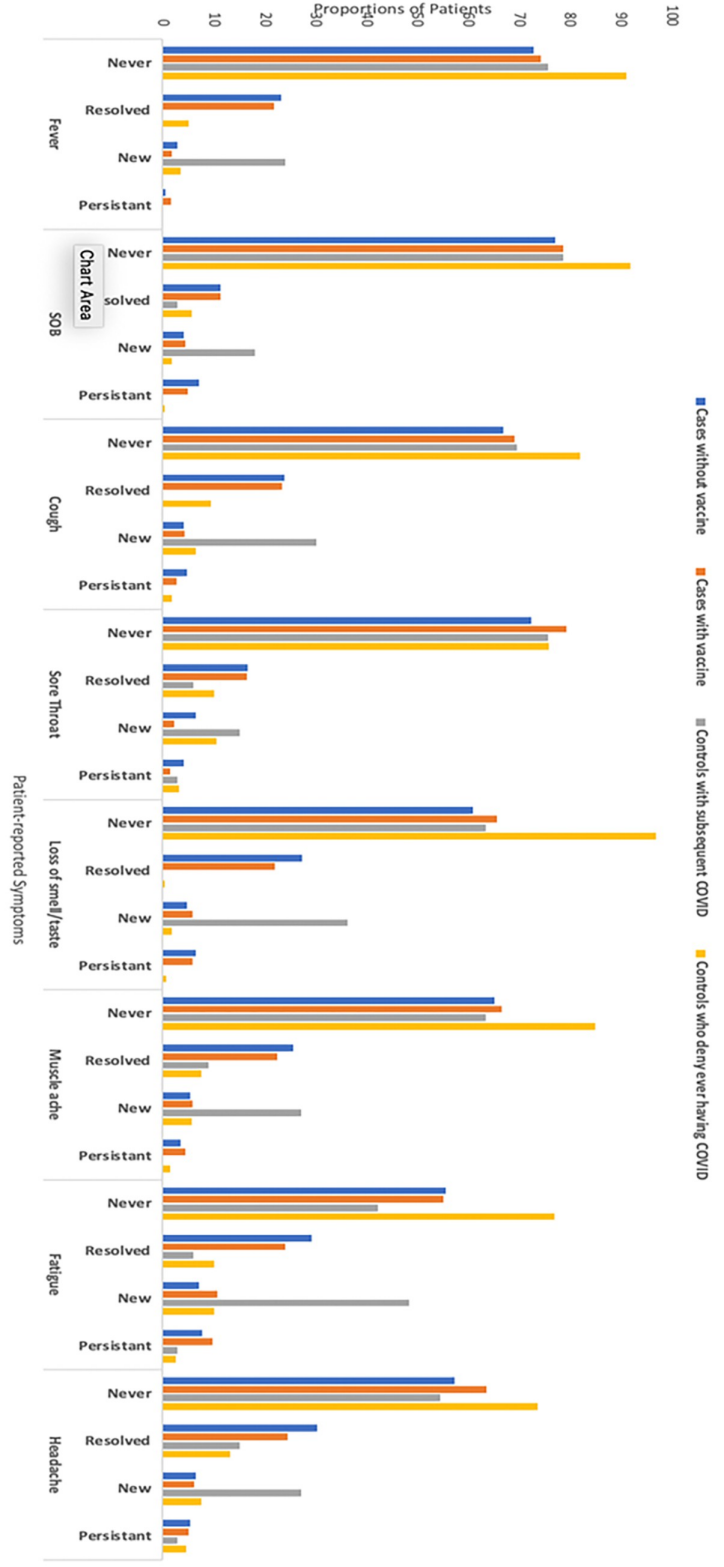
Proportions of patients with potential COVID-19 symptoms.

**Fig 1** depicts the proportion of patients with symptoms on baseline and follow-up questionnaires. During the initial COVID-19 infection, 858 (74.5%) cases reported at least one COVID-19 symptom, whereas 25.5% of cases had asymptomatic infection. The most common COVID-19 symptoms at baseline were fatigue, headache, loss of taste or smell, myalgias, and cough. Although most cases reported improvement in symptoms during follow-up, 21.1% reported having at least one persistent symptom with the most common being persistent shortness of breath, fatigue, and loss of smell/taste (31.6%, 27.6%, and 21.3%, respectively). Among cases with asymptomatic infection at baseline, over 15% of participants reported new fatigue at follow-up, and 8–10% reported new loss of taste/smell, myalgias, or headache. The proportion of cases with new fatigue or loss of taste/smell at follow-up was significantly higher than controls (both p<0.001). Compared to controls, a higher proportion of cases had likely depression (7.1% vs. 2.8%, p<0.001), although there were no differences in likely anxiety (7.6% vs. 6.7%, p = 0.41). Both cases and controls did not report cognitive dysfunction (median CFI 1 and 0, respectively) or functional limitations (median DASI 58.2 for both) during follow-up.

Fourteen controls self-reported subsequent COVID-19 infection between baseline and follow-up questionnaires. These participants reported similar symptoms as cases, including fever (24.2%), shortness of breath (18.2%), cough (30.3%), sore throat (15.2%), loss of smell or taste (36.4%), muscle ache (27.3%), fatigue (48.5%), and headache (27.3%).

**Fig 2:**
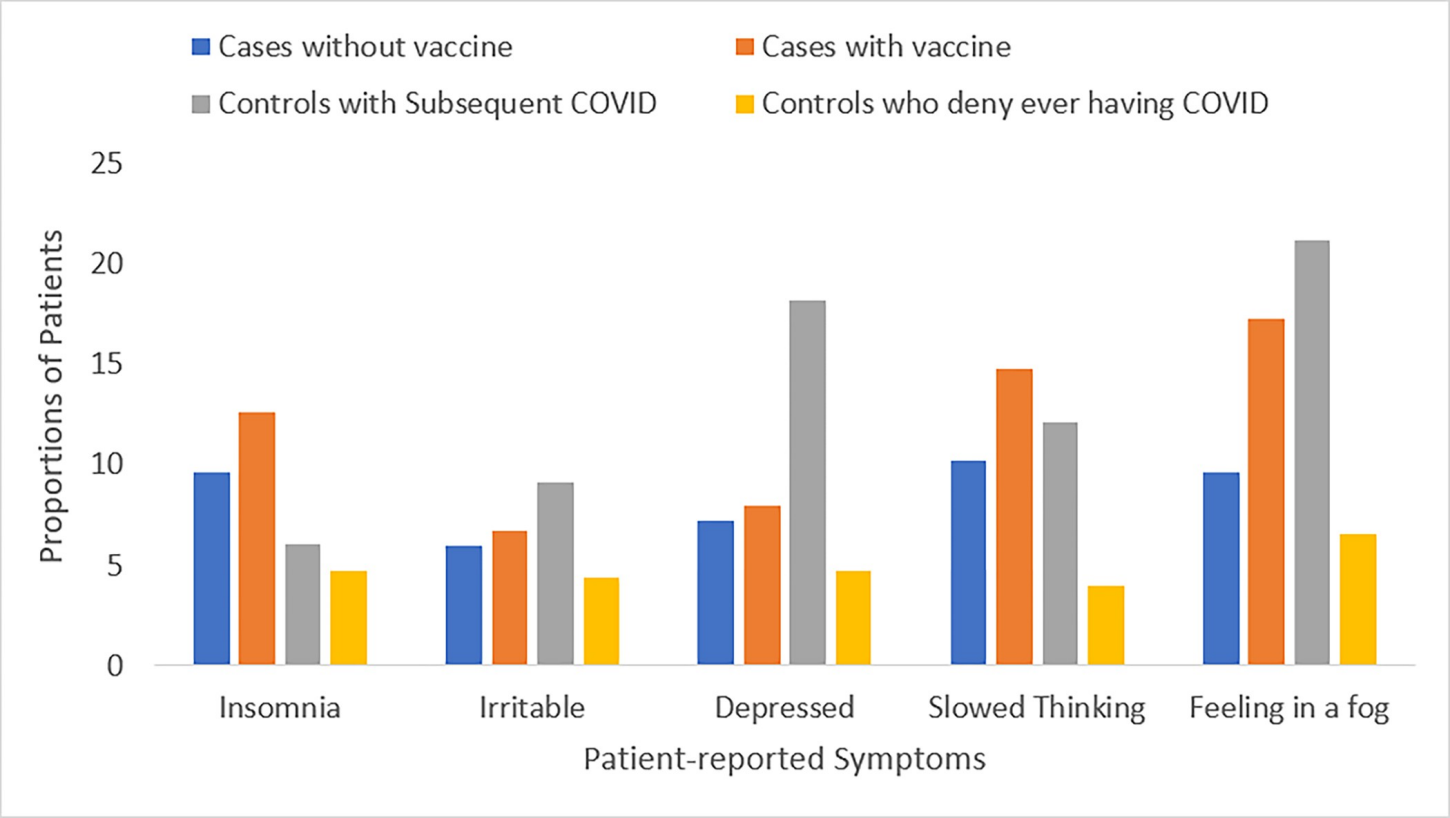
Proportions of patients with potential PASC symptoms*. *Proportions reflect a cross-sectional assessment of symptoms during follow-up as baseline measurements were not available to assess longitudinal changes.

**Fig 2** illustrates commonly reported PASC symptoms that were newly assessed on follow-up questionnaire. Over 10% of cases reported feeling in a fog (16.2%), slowed thinking (14.0%), and insomnia (12.1%); these were similar to proportions observed in controls with subsequent COVID-19 infection (21.2%, 12.1%, and 6.1%, respectively) and higher than controls without subsequent COVID-19 infection (6.6%, 4.0%, and 4.7%, respectively).

### Antibody data and participation

Follow-up antibody testing was completed in 1336 (59.1%) participants, including 723 (62.9%) cases and 613 (55.2%) controls who had completed the follow-up questionnaire. Characteristics of this subgroup are described in **Table 2**. Like the total sample, most participants were 45–64 years old, female, and Non-Hispanic White. Controls continued to be significantly older and had a higher proportion of females, non-Hispanic Whites, and persons with a college degree.

**Table 2:** Characteristics of patients who completed COVID-19 testing.

	Cases (n = 723)	Controls (n = 613)	p-value**
Age 18–24 years 25–44 years 45–64 years 65–89 years	16 (2.2%) 241 (33.3%) 350 (48.4%) 116 (16.0%)	6 (1.0%) 160 (26.1%) 309 (50.4%) 138 (22.5%)	0.001
Sex (% male)	247 (34.2%)	151 (24.6%)	< .001
Race/ethnicity Non-Hispanic White Non-Hispanic Black Hispanic Asian Non-Hispanic other	515 (71.2%) 48 (6.6%) 114 (15.8%) 33 (4.6%) 13 (1.8%)	531 (86.6%) 17 (2.8%) 51 (8.3%) 12 (2.0%) 2 (0.3%)	< .001
Household size 1–2 adults, without children 1–2 adults, with children 3+ adults, without children 3+ adults, with children	366 (50.6%) 182 (25.2%) 93 (12.9%) 71 (9.8%)	372 (60.7%) 136 (22.2%) 70 (11.4%) 31 (5.1%)	0.001
Education Less than high school High school or equivalent Some college College degree	6 (0.8%) 44 (6.1%) 127 (17.6%) 537 (74.3%)	2 (0.3%) 10 (1.6%) 81 (13.2%) 518 (84.5%)	< .001
Socioeconomic status (SES)* Low SES Medium SES High SES	35 (4.8%) 298 (41.2%) 375 (51.9%)	39 (6.4%) 229 (37.4%) 342 (55.8%)	0.17

* Neighborhood socioeconomic status (SES) was assigned based on the estimated proportion of low SES addresses in the county-specific census block group, which was defined as a function of the per capita income and percent of owner-occupied housing units.

** Characteristics of cases and controls were compared using Chi2 tests.

**Fig 3:**
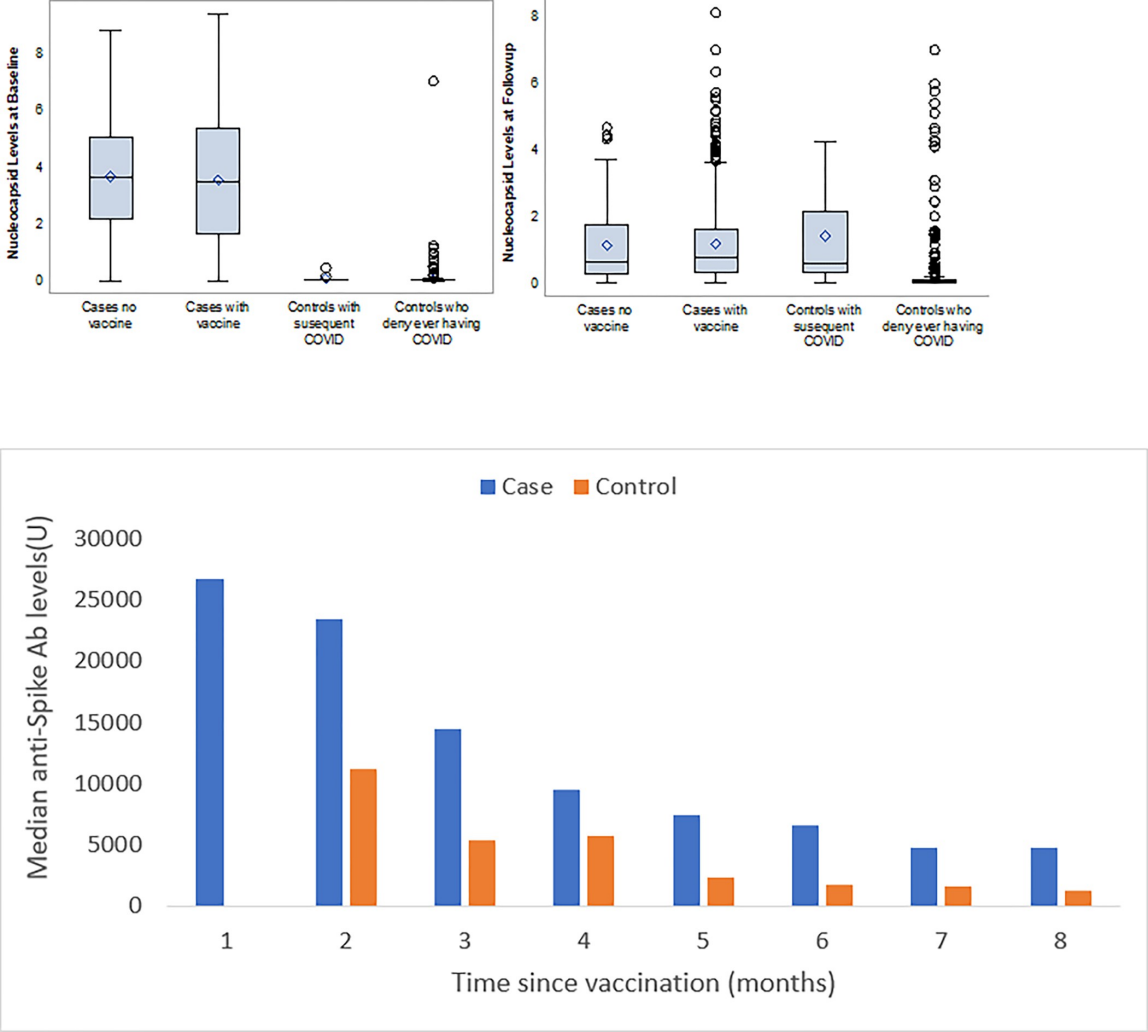
**A.** Anti-nucleocapsid antibody levels during follow-up. **B.** Median anti-spike antibody levels during follow-up after vaccination.

**Fig 3A** summarizes anti-nucleocapsid antibody levels for participants at baseline and follow-up, stratified by case/control status and subsequent vaccination status. The mean time between baseline and follow-up antibody testing was 8.6 months. Among those who completed antibody testing, 84.8% of cases and 97.7% of controls reported being vaccinated between baseline and follow-up testing. At baseline, median nucleocapsid antibody levels for cases with and without COVID-19 vaccination were 3.47 U and 3.66 U, respectively, compared to the median nucleocapsid antibody levels of 0.03 U for both controls with and without vaccination.

At follow-up, median nucleocapsid for cases with and without vaccines decreased to 0.65 U and 0.76 U, respectively. “Sero-reversion” at follow up, with antibody levels below the assay’s positive threshold (< 1.4 U) was observed in 1074 (80.4%) cases. Of these participants 76.8% reported having a mild or asymptomatic infection. Among controls, median nucleocapsid levels remained stable at 0.03 U among those without subsequent COVID-19 infection, whereas controls who reported subsequent COVID-19 infection antibody levels increased to 0.60 U. In adjusted models, there was a statistically significant decrease in anti-nucleocapsid levels among cases (p<0.001) but not controls (p = 0.13).

**Fig 3B** illustrates anti-spike antibodies levels during follow-up after vaccination. Spike antibody levels for both cases and controls differed by months since vaccination. At 4 months following vaccination, median spike antibody levels were only ~50% of those observed in earlier months in cases and controls. Median spike antibody levels among cases continued to be significantly higher than controls; however, median levels for both cases and controls both were below 5000 U by 7 months after vaccination.

## Discussion

In this study among community-dwelling adults, we found more than 1 in 5 participants with COVID-19 infection reported persistent symptoms during follow-up. Further, those with prior COVID-19 infection were more likely than controls to develop new symptoms during follow-up. In fact, over 1 in 7 participants with an asymptomatic infection at baseline developed chronic symptoms suggestive of PASC, such as fatigue, loss of taste/smell, myalgias, and headache. Our results showed that, even among persons with mild COVID-19 infection, it might take months to return to normal health state.

We found that almost 75% of the participants reported symptoms during the acute infection, and of those, 21% had persistent symptoms at follow up (median 8 months). A systematic review reported nearly three-fourths of participants reported persistent symptoms after the acute phase [38]. Consistently, studies reported the persistence of at least one COVID symptom after 4 months of symptom onset, with the most common being fatigue, loss of smell and taste. However, these studies oversampled patients with severe acute infection so may have overestimated the prevalence of PASC symptoms. In our sample, PASC-like symptoms were rarer, albeit still reported by nearly one-fourth of participants, and the most reported persistent symptom was shortness of breath.

Additionally, our study included 25% of cases who did not report any symptoms at baseline; of those, 15% reported at least one new symptom during follow up, mostly symptoms related to mental state, fogginess, and insomnia. A previous analysis among asymptomatic COVID-19 persons similarly showed 30 to 60% of persons reported new symptoms after the initial phase [39]. These data reinforce that the inflammatory response in asymptomatic people can still be substantial even months after the infection [40]. Risk stratification models based on risk factors for PASC, such as female sex, older age, and active smoking may help to identify asymptomatic persons who require more intensive follow up [41]. A multidisciplinary approach to assess symptoms and potential complications as well as provide rehabilitation services may also benefit higher-risk patients and those with PASC.

Describing and understanding the characteristics of antibodies’ response has been an important goal of recent research studies regarding COVID-19 immune response. Although COVID-19 immunity is more complex than antibody levels, higher anti-spike and anti-nucleocapsid antibodies have been associated with both mild and severe infections [42]. Although cases in our study had significantly higher antibody anti-nucleocapsid antibody levels than controls, both anti-nucleocapsid and anti-spike antibody levels appeared to quickly wane over time. Other seroprevalence studies have reported decreasing nucleocapsid and spike protein antibody levels in the months following COVID-19 infection or second vaccine dose [25,26,28,43–45]. In fact, we found up to 80% of the cases in this study had undergone sero-reversion at a relatively short median follow-up of 8 months, with antibody levels below the positive threshold. A previous study found 50% of persons sero-reverted as quickly as 30 days after the first positive PCR result [46]. Severity of clinical illness may influence the duration of the humoral response; in our sample, of those who underwent sero-reversion, 76% reported having a mild or asymptomatic infection. As above, sero-reversion does not necessarily imply lack of any immunity, as T cell immunity still plays a role. Other studies reported T cells react mainly to the spike protein to initiate antiviral immune response [47]. Conversely, T cell perturbations have been reported to persist for several months in several post-viral syndromes and may be associated with PASC after mild COVID-19 infection [48].

Study results should be interpreted considering its limitations. First, we conducted follow-up surveys between June 2021 –December 2021; therefore, recent COVID-19 infection with subsequent variants may have resulted in overestimation of anti-nucleocapsid and anti-spike antibodies, as well as symptoms reported on the follow-up questionnaire. Similarly, we captured timing of initial COVID-19 vaccination but did not collect data on boosters; however, this likely accounted for a minority of participants and would have resulted in an overestimation, not underestimation of anti-spike antibody levels in latter months. Second, surveys are inherently limited by non-response bias, so individuals with persistent symptoms, repeat infection, or interest in COVID-19 immunity may have been more likely to participate. The risk of non-response bias was highlighted by 90% of participants having a history of COVID-19 vaccine–significantly higher than vaccination uptake in the general population. Third, response bias may have resulted from participants responding in a socially desirable manner rather than their true symptoms. These limitations are outweighed by the study’s strengths including its community-based sampling, inclusion of persons with mild COVID-19 infection at baseline and pairing of longitudinal surveys with antibody testing.

Overall, mild, and asymptomatic cases represent a large faction of all COVID-19 cases in the general population. Data regarding the risk and long-term clinical consequences of PASC, as well as immune responses after COVID-19 infection and vaccination, are necessary to prepare for remaining challenges facing healthcare systems and to coordinate the multidisciplinary care that this group may require.

## Supporting information

S1 File (PDF)
