# Supplementary material for: Post-acute sequelae of COVID-19 and longitudinal antibody levels in a community-based cohort
Source: PLoS One. 2023 Sep 8;18(9):e0291259. doi: 10.1371/journal.pone.0291259 (PMC10490864; doi:10.1371/journal.pone.0291259)
Supplement: S1 File — (PDF) [file pone.0291259.s001.pdf]

DFW COVID-19 Prevalence Study

MRN: ______________________________

We appreciate your participation in the DFW COVID-19 Prevalence Study to help us better understand the impact of COVID-19 on our Dallas and Tarrant county communities. This study is being conducted by the University of Texas Southwestern Medical Center and Texas Health Resources. Your participation and answers are confidential.

After you complete the survey, a member of our team will contact you to schedule your COVID antibody testing. If you have any questions, please contact the research team at 214-648-5005.

Apreciamos su participación en el Estudio de Prevalencia de COVID-19 en DFW para ayudarnos a entender mejor el impacto de COVID-19 en nuestras comunidades de los Condados de Dallas y Tarrant. Este estudio se está llevando a cabo por University of Texas Southwestern Medical Center y Texas Health Resources. Su participación y respuestas son confidenciales.

Después de que complete la encuesta, un miembro de nuestro equipo se comunicará con usted para programar su prueba de anticuerpos de COVID. Si tiene alguna pregunta, por favor comuníquese con el equipo de investigación al 214-648-5005.

What language do you prefer?
¿Qué idioma prefiere?

The survey will begin upon making this selection.
La encuesta comenzará al hacer esta selección.

- ◯ English/Inglés
- ◯ Spanish/Español

Since your study participation on [appt_date] have you been tested for COVID-19 again?

- ◯ Yes - one time
- ◯ Yes - more than one time
- ◯ No

Desde su participación en el estudio en [appt_date], ¿se ha hecho la prueba de COVID-19 otra vez?

- ◯ Sí - una vez
- ◯ Sí - más de una vez
- ◯ No

What month were you tested? If you have been tested more than once, please enter the month of your most recent test.

- ◯ September 2020
- ◯ October 2020
- ◯ November 2020
- ◯ December 2020
- ◯ January 2021
- ◯ February 2021
- ◯ March 2021
- ◯ April 2021
- ◯ May 2021
- ◯ June 2021
- ◯ July 2021
- ◯ August 2021
- ◯ September 2021
- ◯ October 2021
- ◯ November 2021

¿En qué mes le hicieron la prueba? Si le han hecho la prueba más de una vez, por favor ingrese el mes de su prueba más reciente.	◯ Septiembre de 2020<br>◯ Octubre de 2020<br>◯ Noviembre de 2020<br>◯ Diciembre de 2020<br>◯ Enero de 2021<br>◯ Febrero de 2021<br>◯ Marzo de 2021<br>◯ Abril de 2021<br>◯ Mayo de 2021<br>◯ Junio de 2021<br>◯ Julio de 2021<br>◯ Agosto de 2021<br>◯ Septiembre de 2021<br>◯ Octubre de 2021<br>◯ Noviembre de 2021
What were the results of these COVID-19 test(s) after study participation?	◯ I had COVID-19<br>◯ I did NOT have COVID-19<br>◯ The test could not determine if I had COVID-19 or I did not get my results
¿Cuál fue el resultado de esta(s) prueba(s) de COVID-19 después de la participación en el estudio?	◯ Yo tenía COVID-19<br>◯ Yo NO tenía COVID-19<br>◯ La prueba no pudo determinar si yo tenía COVID-19, o no recibí mis resultados
Have you received the COVID-19 vaccine?	◯ No<br>◯ Yes, I received the COVID-19 vaccine
¿Ha recibido la vacuna contra el COVID-19?	◯ No<br>◯ Sí, recibí la vacuna contra el COVID-19
What COVID-19 vaccine did you receive?	◯ Moderna<br>◯ Pfizer<br>◯ AstraZeneca<br>◯ Johnson and Johnson (J&J or Janssen)<br>◯ Other<br>◯ I don't know
¿Qué vacuna contra el COVID-19 recibió?	◯ Moderna<br>◯ Pfizer<br>◯ AstraZeneca<br>◯ Johnson and Johnson (J&J o Janssen)<br>◯ Otra<br>◯ No sé
When did you receive your first dose of COVID-19 vaccine?	◯ In a clinical trial before December 2020<br>◯ December 2020<br>◯ January 2021<br>◯ February 2021<br>◯ March 2021<br>◯ April 2021<br>◯ May 2021<br>◯ June 2021<br>◯ July 2021<br>◯ August 2021<br>◯ September 2021<br>◯ October 2021<br>◯ November 2021

¿Cuándo recibió la primera dosis de la vacuna contra el COVID-19?	◯ En un ensayo clínico antes de diciembre de 2020<br>◯ Diciembre de 2020<br>◯ Enero de 2021<br>◯ Febrero de 2021<br>◯ Marzo de 2021<br>◯ Abril de 2021<br>◯ Mayo de 2021<br>◯ Junio de 2021<br>◯ Julio de 2021<br>◯ Agosto de 2021<br>◯ Septiembre de 2021<br>◯ Octubre de 2021<br>◯ Noviembre de 2021
Do you plan to take the COVID-19 vaccine when offered?	◯ Very unlikely<br>◯ Unlikely<br>◯ Undecided<br>◯ Likely<br>◯ Very likely
¿Planea ponerse la vacuna contra el COVID-19 cuando sea ofrecida?	◯ Muy improbable<br>◯ Improbable<br>◯ Indeciso(a)<br>◯ Probable<br>◯ Muy probable
Why would you NOT get a COVID-19 vaccine?	☐ I'm allergic to vaccines.<br>☐ I don't like needles.<br>☐ I'm not concerned about getting really sick from COVID-19.<br>☐ I'm concerned about side effects from the COVID-19 vaccine.<br>☐ I don't think COVID-19 vaccines work very well.<br>☐ I don't trust that the COVID-19 vaccine will be safe long-term.<br>☐ I don't believe the COVID-19 pandemic is as bad as some people say it is.<br>☐ I don't know enough about how well a COVID-19 vaccine works.<br>☐ Other, please specify: ______<br>(Please select all that apply)
¿Por qué NO se pondría la vacuna contra el COVID-19?	☐ Soy alérgico(a) a las vacunas<br>☐ No me gustan las agujas<br>☐ No me preocupa enfermarme mucho de COVID-19.<br>☐ Me preocupan los efectos secundarios de la vacuna contra el COVID-19.<br>☐ No creo que las vacunas contra el COVID-19 funcionen muy bien.<br>☐ No confío en que la vacuna contra el COVID-19 será segura a largo plazo.<br>☐ No creo que la pandemia de COVID-19 sea tan mala como dicen algunas personas.<br>☐ No sé lo suficiente sobre qué tan bien funciona una vacuna contra el COVID-19.<br>☐ Otra, por favor especifique: ______

Have you had any of the following symptoms (new or worse compared to one year ago)?	☐ Fever ______<br>☐ Shortness of breath or trouble breathing ______<br>☐ Cough ______<br>☐ Sore throat ______<br>☐ Loss of smell or taste ______<br>☐ Muscle aches or body aches ______<br>☐ Fatigue ______<br>☐ Headaches ______<br>☐ Dizziness ______<br>☐ Difficulty sleeping ______<br>☐ Being irritable ______<br>☐ Feeling depressed or tearful ______<br>☐ Slowed thinking ______<br>☐ Feeling in a "fog" ______<br>☐ No symptoms
¿Ha tenido alguno de los siguientes síntomas (nuevos o peores comparado a un año atrás	☐ Fiebre ______<br>☐ Falta de aire o dificultad para respirar ______<br>☐ Tos ______<br>☐ Dolor de garganta ______<br>☐ Pérdida del olfato o del gusto ______<br>☐ Dolores musculares o del cuerpo ______<br>☐ Fatiga ______<br>☐ Dolores de cabeza ______<br>☐ Mareo ______<br>☐ Dificultad para dormir ______<br>☐ Estar irritable ______<br>☐ Sentirse deprimido(a) o lloroso(a) ______<br>☐ Pensamiento lento ______<br>☐ Sentirse aturdido(a) ______<br>☐ No síntomas
Since study participation, have you ever been told by a doctor or other healthcare professional that you developed any of the following? Please select all that apply.	☐ Heart disease<br>☐ Lung disease<br>☐ Ulcer or stomach disease<br>☐ Liver disease<br>☐ Cancer<br>☐ HIV<br>☐ Stroke<br>☐ Currently pregnant<br>☐ Auto-immune disease<br>☐ High blood pressure<br>☐ Diabetes<br>☐ Kidney disease<br>☐ Anemia or other blood disease<br>☐ Rheumatoid arthritis<br>☐ Neurologic (brain) disease (e.g., dementia)<br>☐ Obesity<br>☐ Immunocompromised, please specify ______<br>☐ Other, please specify ______<br>☐ None of the above

Desde la participación en el estudio, ¿alguna vez le ha dicho un doctor u otro profesional de la salud que usted desarrolló alguno de los siguientes? Por favor seleccione todos los que apliquen.	☐ Enfermedad del corazón<br>☐ Enfermedad pulmonar<br>☐ Úlcera o enfermedad estomacal<br>☐ Enfermedad del hígado<br>☐ Cáncer<br>☐ VIH<br>☐ Derrame cerebral<br>☐ Actualmente embarazada<br>☐ Enfermedad autoinmune<br>☐ Alta presión<br>☐ Diabetes<br>☐ Enfermedad de los riñones<br>☐ Anemia u otra enfermedad de la sangre<br>☐ Artritis reumatoide<br>☐ Enfermedad neurológica (cerebral) (por ejemplo, demencia)<br>☐ Obesidad<br>☐ Inmunocomprometido(a), por favor especifique ______<br>☐ Otro, por favor especifique ______<br>☐ Ninguna de las anteriores
During your COVID infection, did you experience any COVID-related symptoms? COVID symptoms include fever, shortness of breath/trouble breathing, cough, sore throat, loss of smell or taste, muscle/body aches.	◯ Yes<br>◯ No<br>◯ Don't recall
Durante su infección de COVID, ¿experimentó algún síntoma relacionado con COVID? Los síntomas de COVID incluyen fiebre, falta de aire/dificultad para respirar, tos, dolor de garganta, pérdida del olfato o del gusto, dolores musculares/corporales.	◯ Sí<br>◯ No<br>◯ No recuerdo
Were the symptoms severe enough that you went to the emergency department or were admitted to the hospital?	◯ Yes, I went to the emergency department but was not admitted to the hospital<br>◯ Yes, I was admitted to the hospital<br>◯ No
¿Fueron los síntomas lo suficientemente severos que fue a la sala de emergencias o fue admitido(a) en el hospital?	◯ Sí, fui a la sala de emergencias, pero no me admitieron en el hospital<br>◯ Sí, fui admitido(a) en el hospital<br>◯ No
How long did it take for you to return to your normal state of health?	◯ Within one week<br>◯ Longer than one week but within one month<br>◯ More than one month but within three months<br>◯ More than three months but within 6 months<br>◯ More than 6 months<br>◯ I still feel like I am not back to my usual state of health
¿Cuánto tiempo le tomó para regresar a su estado normal de salud?	◯ En una semana<br>◯ Más de una semana, pero menos de un mes<br>◯ Más de un mes, pero menos de tres meses<br>◯ Más de tres meses, pero menos de 6 meses<br>◯ Más de 6 meses<br>◯ Aún siento como que no he regresado a mi estado normal de salud

If you are not back at usual state of health, what symptoms are you still experiencing?	☐ Shortness of breath or trouble breathing<br>☐ Cough<br>☐ Sore throat<br>☐ Loss of smell or taste<br>☐ Muscle aches or body aches<br>☐ Fatigue<br>☐ Headaches<br>☐ Dizziness<br>☐ Difficulty sleeping<br>☐ Being irritable<br>☐ Feeling depressed or tearful<br>☐ Slowed thinking<br>☐ Other, please specify ______
Si no ha regresado a su estado normal de salud, ¿qué síntomas aún está experimentando?	☐ Falta de aire o dificultad para respirar<br>☐ Tos<br>☐ Dolor de garganta<br>☐ Pérdida del olfato o del gusto<br>☐ Dolores musculares o del cuerpo<br>☐ Fatiga<br>☐ Dolores de cabeza<br>☐ Mareo<br>☐ Dificultad para dormir<br>☐ Estar irritable<br>☐ Sentirse deprimido(a) o lloroso(a)<br>☐ Pensamiento lento<br>☐ Otro, por favor especifique ______
Please describe the nature of your symptoms	◯ Symptoms have been constant without any change over time<br>◯ Symptoms have slowly been improving over time<br>◯ Symptoms have slowly been getting worse over time<br>◯ Symptoms come and go at different times and seem unpredictable<br>◯ I had new "long-hauler" symptoms start after my COVID-19 infection had resolved<br>◯ Other, please specify ______
Por favor caracterice sus síntomas	◯ Síntomas han sido constantes sin ningún cambio con el paso del tiempo<br>◯ Síntomas han estado mejorando lentamente con el paso del tiempo<br>◯ Síntomas han estado empeorando lentamente con el paso del tiempo<br>◯ Síntomas vienen y van en diferentes momentos y parecen impredecibles<br>◯ Tuve nuevos síntomas de "largo plazo" que empezaron después de que mi infección de COVID-19 se había resuelto<br>◯ Otro, por favor especifique ______

Over the past 2 weeks, how often you been bothered by following problems?

	Not at all	Several days	More than half the days	Nearly every day
Little interest or pleasure in doing things	◯	◯	◯	◯
Feeling down, depressed, or hopeless	◯	◯	◯	◯
Feeling nervous, anxious, or on edge	◯	◯	◯	◯
Not being able to stop or control worrying	◯	◯	◯	◯

En las últimas 2 semanas, ¿qué tan seguido le han molestado los siguientes problemas?

	Ningún día	Varios días	Más de la mitad de los días	Casi todos los días
Poco interés o placer en hacer cosas	◯	◯	◯	◯
Sentirse decaído(a), deprimido(a) o sin esperanzas	◯	◯	◯	◯
Sentirse nervioso(a), ansioso(a), o tenso(a)	◯	◯	◯	◯
No ser capaz de parar o controlar su preocupación	◯	◯	◯	◯

Please indicate how true each statement has been for you during the past 7 days.

	Not at all	A little bit	Somewhat	Quite a bit	Very much
I feel fatigued	◯	◯	◯	◯	◯
I feel weak all over	◯	◯	◯	◯	◯
I feel listless (washed out)	◯	◯	◯	◯	◯
I feel tired	◯	◯	◯	◯	◯
I have trouble starting things because I am tired	◯	◯	◯	◯	◯
I have trouble finishing things because I am tired	◯	◯	◯	◯	◯
I have energy	◯	◯	◯	◯	◯
I am able to do my usual activities	◯	◯	◯	◯	◯
I need to sleep during the day	◯	◯	◯	◯	◯
I am too tired to eat	◯	◯	◯	◯	◯
I need help doing my usual activities	◯	◯	◯	◯	◯
I am frustrated by being too tired to do the things I want to do	◯	◯	◯	◯	◯

I have to limit my social activity because I am tired	◯	◯	◯	◯	◯

Por favor marque un solo número por línea para indicar la respuesta que corresponde a los últimos 7 días.

	Nada	Un poco	Algo	Mucho	Muchísimo
Me siento agotado(a)	◯	◯	◯	◯	◯
Siento debilidad en todo el cuerpo	◯	◯	◯	◯	◯
Me siento decaído(a)	◯	◯	◯	◯	◯
Me siento cansado(a)	◯	◯	◯	◯	◯
Tengo dificultad para comenzar las cosas porque estoy cansado(a)	◯	◯	◯	◯	◯
Tengo dificultad para terminar las cosas porque estoy cansado(a)	◯	◯	◯	◯	◯
Tengo energía	◯	◯	◯	◯	◯
Puedo hacer mis actividades usualesSoy capaz de hacer mis actividades habituales (p. ej., trabajar, ir a la escuela, hacer las compras)	◯	◯	◯	◯	◯
Necesito dormir durante el día	◯	◯	◯	◯	◯
Estoy demasiado cansado(a) para comer	◯	◯	◯	◯	◯
Necesito ayuda para hacer mis actividades habituales	◯	◯	◯	◯	◯
Estoy frustrado(a) porque estoy demasiado cansado(a) para hacer las cosas que quiero hacer	◯	◯	◯	◯	◯
Tengo que limitar mis actividades sociales debido al cansancio	◯	◯	◯	◯	◯

Please answer all questions compared to one year ago.

	Yes	No	Maybe
Do you feel your memory has declined substantially?	◯	◯	◯
Do others tell you that you tend to repeat questions over and over?	◯	◯	◯
Have you been misplacing things more often?	◯	◯	◯

Do you find that lately you are relying more on written reminders?	○	○	○
Do you need more help from others to remember appointments, family occasions, or holidays?	○	○	○
Do you have more trouble recalling names, finding the right word, or completing sentences?	○	○	○
Do you have more trouble driving (e.g., do you drive more slowly, have more trouble at night, tend to get lost, have accidents)?	○	○	○
Do you have more difficulty managing money (e.g. paying bills, calculating change, completing tax forms)?	○	○	○
Are you less involved in social activities?	○	○	○
Has you work performance (paid or volunteer) declined significantly compared to one year ago?	○	○	○
Do you have more trouble following the news, or the plots of books, movies or TV shows compared to one year ago?	○	○	○
Are there any activities (e.g. hobbies, such as card games, crafts) that are substantially more difficult for you now compared to one year ago?	○	○	○
Are you more likely to become disoriented, or get lost, for example when traveling to another city?	○	○	○
Do you have more difficulty using household appliances (such as the washing machine, television remote, DVD, or computer)?	○	○	○

Por favor responda todas las preguntas comparando con hace un año.

	Sí	No, podía hacer esto hace 1 año atrás pero estoy teniendo dificultad ahora	No, no he podido hacer esto en más de 1 año
Por favor responda todas las preguntas comparando con hace un año.	○	○	○
¿Otros le dicen que suele repetir preguntas una y otra vez?	○	○	○
¿Ha estado perdiendo cosas más seguido?	○	○	○
¿Encuentra que recientemente está dependiendo más en recordatorios escritos?	○	○	○
¿Necesita más ayuda de otros para recordar citas, ocasiones familiares, o días festivos?	○	○	○
¿Tiene más dificultad recordando nombres, encontrando la palabra correcta, o completando oraciones?	○	○	○
¿Tiene más dificultad manejando (p. ej., maneja más lento, tiene más dificultad en la noche, suele perderse, tiene accidentes)?	○	○	○
¿Tiene más dificultad administrando dinero (p. ej. pagando facturas, calculando cambio, completando formularios de impuestos)?	○	○	○
¿Está menos involucrado en actividades sociales?	○	○	○
¿Ha disminuido significativamente su desempeño laboral (pagado o voluntario) comparado a un año atrás?	○	○	○
¿Tiene más dificultad siguiendo las noticias, o las tramas de libros, películas o programas de televisión comparado a un año atrás?	○	○	○

¿Hay alguna actividad (p. ej. pasatiempos, tales como juegos de cartas, manualidades) que es considerablemente más difícil para usted ahora comparado a un año atrás?	○	○	○
¿Es más probable que usted se desoriente, o se pierda, por ejemplo al viajar a otra ciudad?	○	○	○
¿Tiene más dificultad usando electrodomésticos (tales como la lavadora, el control remoto de la televisión, DVD, o computadora)?	○	○	○

Can you do the following behaviors?

	Yes	No, I was able to do 1 year ago but having difficulty now	No, I have not been able to do this for more than 1 year
Take care of yourself, that is, eat, bathe, and use the toilet?	○	○	○
Walk indoors, such as around your house?	○	○	○
Walk a block or two on level ground?	○	○	○
Climb a flight of stairs or walk up a hill?	○	○	○
Run a short distance?	○	○	○
Do light work around the house like dusting or washing dishes?	○	○	○
Do moderate work around the house like vacuuming, sweeping floors, and carrying in groceries?	○	○	○
Do heavy work around the house like scrubbing floors or lifting or moving heavy furniture?	○	○	○
Do garden work like raking leaves, weeding, or pushing lawn mower?	○	○	○
Have sexual relations?	○	○	○
Participate in moderate recreational activities like golf, bowling, dancing, doubles tennis, or throwing a ball?	○	○	○

Participate in strenuous sports like swimming, singles tennis, football, basketball, or skiing?	○	○	○

¿Puede realizar los siguientes comportamientos?

	Sí	No, podía hacer esto hace 1 año atrás pero estoy teniendo dificultad ahora	No, no he podido hacer esto en más de 1 año
¿Cuidarse, es decir, comer, bañarse, y usar el baño?	○	○	○
¿Caminar dentro, tal como en su casa?	○	○	○
¿Caminar una cuadra o dos en suelo plano?	○	○	○
¿Subir un tramo de escaleras o subir una colina?	○	○	○
¿Correr una distancia corta?	○	○	○
¿Hacer trabajo ligero alrededor de la casa como desempolvar o lavar los platos?	○	○	○
¿Hacer trabajo moderado en la casa como aspirar, barrer los pisos, y llevar las bolsas de alimentos adentro?	○	○	○
¿Hacer trabajo pesado en la casa como fregar el piso o levantando o moviendo muebles pesados?	○	○	○
¿Hacer trabajo de jardinería como juntar hojas, deshierbar, o empujar la cortadora de pasto?	○	○	○
¿Tener relaciones sexuales?	○	○	○
¿Participar en actividades recreacionales moderadas como golf, boliche, baile, tenis en dobles, o lanzar una pelota?	○	○	○
¿Participar en deportes arduos como natación, tenis de individuales, fútbol americano, baloncesto, o esquiar?	○	○	○

Please provide your contact information and preferred communication method.

First Name ______
Last Name ______
Address ______
Email ______
Phone Number ______

Please provide your contact information and preferred communication method.

Primer Nombre:

______
Apellido: ______
Dirección:

______
Correo Electrónico:

______
Número de Teléfono:

______

What is your preferred method for us to contact you?	◯ Email<br>◯ Phone
¿Cuál es su método preferido para contactarle?	◯ Correo Electrónico<br>◯ Teléfono
